# Antifungal Nafuredin and Epithiodiketopiperazine Derivatives From the Mangrove-Derived Fungus *Trichoderma harzianum* D13

**DOI:** 10.3389/fmicb.2020.01495

**Published:** 2020-06-26

**Authors:** Dong-Lin Zhao, Xi-Fen Zhang, Rui-Huan Huang, Dan Wang, Xiao-Qiang Wang, Yi-Qiang Li, Cai-Juan Zheng, Peng Zhang, Cheng-Sheng Zhang

**Affiliations:** ^1^Tobacco Research Institute, Chinese Academy of Agricultural Sciences, Qingdao, China; ^2^Plant Protection Station of Shandong Province, Jinan, China; ^3^Key Laboratory of Tropical Medicinal Resource Chemistry of Ministry of Education, Hainan Normal University, Haikou, China

**Keywords:** nafuredins, epithiodiketopiperazines, *Magnaporthe oryzae*, antifungal activity, *Trichoderma harzianum*

## Abstract

A new polyketide derivative, nafuredin C (**1**), a novel heterocyclic dipeptide, trichodermamide G (**3**), together with four known biogenetically related compounds nafuredin A (**2**), trichodermamide A (**4**), aspergillazin A (**5**), and peniisocoumarin H (**6**), were isolated from the mangrove-derived fungus *Trichoderma harzianum* D13. Their structures, including their absolute configurations, were determined by spectroscopic analysis and time-dependent density functional theory-electronic circular dichroism (ECD) calculations. Trichodermamide G was found to be a novel epithiodiketopiperazine derivative with an unprecedented cyclic system containing a sulfur bridge, and nafuredin C represented the third nafuredin derivative of these homologous compounds. The new compound nafuredin C exhibited obvious antifungal activity against *Magnaporthe oryzae* with a minimum inhibitory concentration (MIC) of 8.63 μM, which is on the same order of magnitude as the positive control carbendazim (MIC = 3.27 μM).

## Introduction

Rice blast disease is the most serious disease affecting cultivated rice, a staple food for nearly 50% of the world’s population, and seriously threatens global food security. Each year, rice blast disease is responsible for the loss of 10–30% of the rice harvest, which is enough to feed more than 60 million people (Martin-Urdiroz et al., 2016; Zhou, 2016). Rice blast is caused by *Magnaporthe oryzae*, an ascomycete fungus that causes diseases in a wide range of economically important crops including barley, oats, rye grass, and millets. It was first reported in Brazil in 1985 and then rapidly spread to other South American countries, causing significant yield losses. The disease captured public attention in 2016 when it appeared in Bangladesh, resulting in thousands of rice fields being burned to prevent further spread of the disease (Ma and Xu, 2019). In China, rice blast occurs everywhere rice is cultivated, particularly in hilly areas. In epidemic years of the disease, yield loss can reach 40–50% and in severe cases can result in complete losses in major rice production areas (Zhao X. X. et al., 2019). Therefore, searching for new biological pesticides to control rice blast is necessary for sustainable development of the rice industry and food security worldwide.

Beneficial microbes can function as biocontrol organisms that help plants defend themselves from attack by pathogens. Fungi in the genus *Trichoderma* are excellent mycoparasites of plant pathogens and directly protect plants against them. In addition, *Trichoderma* spp. can enhance the plant defense system, which enables the plant to respond in a fast and strong manner to pathogen attack (Guzman-Guzman et al., 2019). These fungi are also prolific producers of numerous secondary metabolites with pharmaceutical and biotechnological importance, including polyketides, non-ribosomal peptides, siderophores, peptaibols, and volatile and non-volatile terpenes (Contreras-Cornejo et al., 2016). Several studies have focused on the application of *Trichoderma* spp. for controlling rice blast disease with apparent effects; however, marine-derived *Trichoderma* spp. have not been evaluated for use in plant protection, and few studies have reported their antifungal effects against *M. oryzae*.

**FIGURE 1:**
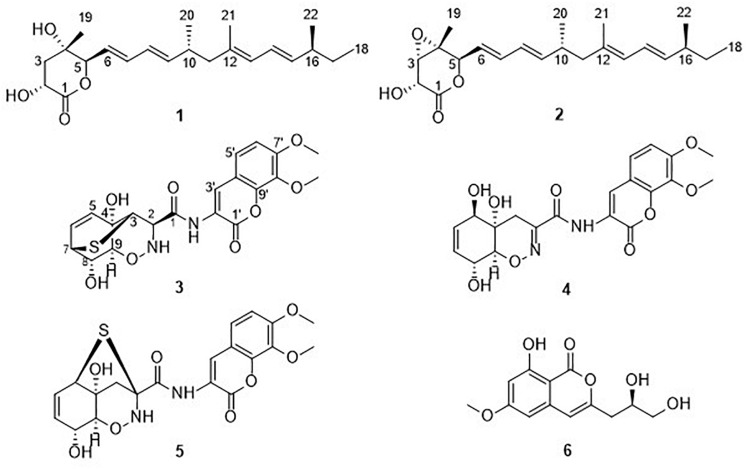
Chemical structures of compounds **1–6**.

During our ongoing search for new anti-phytopathogenic fungal secondary metabolites from marine-derived fungi in the Yellow Sea and South China Sea (Huang et al., 2018; Zhao et al., 2018; Zhao D. L. et al., 2019), we found that the extract of mangrove-derived fungus *Trichoderma harzianum* D13, collected from Hainan province, China, displayed a strong activity against fungal plant pathogens. Further chemical investigation of the ethyl acetate (EtOAc) extracts led to the isolation of six compounds (Figure 1), including two nafuredin derivatives, nafuredins C and A (**1** and **2**); three epithiodiketopiperazine derivatives, trichodermamide G (**3**), trichodermamide A (**4**), and aspergillazin A (**5**); and one isocoumarin, peniisocoumarin H (**6**). Among them, nafuredin C and trichodermamide G are new compounds. Herein, we report the isolation, structural elucidation, and antifungal activities of these compounds.

## Materials and Methods

### General Experimental Procedures

Specific rotations were measured on a JASCO P-1020 digital polarimeter (Tokyo, Japan). ECD spectra were obtained on a JASCO J-815-150S spectropolarimeter. UV spectra were recorded on a Techcomp UV2310II spectrophotometer (Shanghai, China). The 1D (^1^H, ^13^C, and NOE) and 2D NMR spectra [HMQC, correlation spectroscopy (COSY), heteronuclear multiple bond correlation (HMBC), and nuclear Overhauser effect spectroscopy (NOESY)] were acquired on a DD2 500 MHz NMR spectrometer (Agilent Technologies, Santa Clara, CA, United States). A Micromass Q-TOF spectrometer (Waters, Milford, MA, United States) and Thermo Scientific LTQ Orbitrap XL spectrometer (Waltham, MA, United States) was used to measure the electrospray ionization mass spectrometry (ESIMS) and HRESIMS. High-performance liquid chromatography (HPLC) was performed using a Waters e2695 separation module with a Waters 2998 detector and Waters X-Bridge C_18_ (5 μm, 10 × 250 mm) column. Column chromatography (CC) was performed over silica gel (200–300 mesh; Qing Dao Hai Yang Chemical Group Co., Qingdao, China), octadecylsilyl silica (ODS) gel (RP18, 40–63 μm; Merck, Billerica, MA, United States), and Sephadex LH-20 (GE Healthcare, Little Chalfont, United Kingdom). Precoated silica gel plates (G60, F-254; Yan Tai Zi Fu Chemical Group Co., Yantai, China) were used for analytical thin-layer chromatography. Analytical and HPLC-grade solvents were used for isolation.

### Fungal Material

The fungus was isolated from the internal tissues of the root of mangrove plant *Excoecaria agallocha* Linn. collected from Hainan province, China, in 2016, and was identified as *T. harzianum* (GenBank accession number MG827165) by sequence analysis of the internal transcribed spacer region of the rDNA. A voucher strain of this fungus was deposited in the Marine Agriculture Research Center, Tobacco Research Institute of Chinese Academy of Agricultural Sciences, Qingdao, China.

### Extraction and Isolation

The fungus *T. harzianum* D13 was cultured on plates of potato dextrose agar medium at 28°C for 3 days. Plugs of agar supporting mycelium growth were cut and transferred aseptically into 200 × 1000-mL Erlenmeyer flasks each containing 400 mL of potato dextrose water liquid medium. The flasks were incubated at 28°C under static conditions for 30 days. The cultures (80 L) were filtered through gauze to separate the mycelial layer from the aqueous layer. The filtrate was then extracted twice with EtOAc, whereas the mycelium were mechanically broken and ultrasonically disrupted for 10 min and then extracted twice with CH_2_Cl_2_:MeOH (1:1, v/v). After removing CH_2_Cl_2_ and MeOH by evaporation under vacuum, the remaining aqueous solution was extracted three times with EtOAc. The combined EtOAc extracts were concentrated under reduced pressure to yield the total EtOAc extract (13.8 g). This extract was subjected to vacuum liquid chromatography on silica gel for elution with a gradient of EtOAc in petroleum ether (EtOAc/petroleum ether, 0–100%), and then with MeOH in EtOAc ranging from 10 to 50% to give eight fractions (Fractions 1–8). Fraction 3 was initially fractionated using an ODS gel column with a step gradient elution of MeOH–H_2_O (60–90%) to afford Fr. 3-1 and Fr. 3-2. Fraction 3-1 was subsequently applied to a Sephadex LH-20 CC (CH_2_Cl_2_/MeOH, v/v, 1/1), and finally purified by reversed-phase-HPLC eluting with 80% MeOH–H_2_O to obtain compound **2** (10.6 mg). Fraction 4 was separated on an ODS column eluting with 50–90% MeOH–H_2_O to obtain Fr. 4-1 and Fr. 4-2. Fraction 4-1 was then afforded to silica gel CC (EtOAc/petroleum ether, 5% to 35%), followed by purification by HPLC (MeOH/H_2_O, 80/20) to yield **1** (10.0 mg). Fraction 6 was subjected to ODS CC using a gradient elution of 30–90% MeOH–H_2_O, followed by separation on Sephadex LH-20 (CH_2_Cl_2_–MeOH, v/v, 1/1) to afford subfractions Fr. 6-1–6-3. Fraction 6-1 was subjected to silica gel CC (MeOH/CH_2_Cl_2_, v/v, 1/50 to 1/10), and finally purified by semipreparative HPLC eluting with 35% MeCN–H_2_O to give **6** (4.0 mg). Fractions 6-2–6-4 were purified by HPLC using 40, 40, and 35% MeOH–H_2_O to give **5** (9.0 mg), **4** (68.5 mg), and **3** (32.0 mg), respectively.

Nafuredin C (**1**): white, amorphous powder; [*α*]^20^_D_ +40.0 (*c* 0.37, MeOH); UV (MeOH) *λ*_max_ (log *ε*) 240 (4.38) nm; ECD (0.93 mM, MeOH) *λ*_max_ (Δ*ε*) 222 (-17.40), 244 (+21.99) nm; ^1^H and ^13^C NMR data, see Table 1; HRESIMS *m/z* 385.2349 [M +Na]^+^ (calculated for C_22_H_38_O_4_Na, 380.2795).

**TABLE 1:** ^1^H NMR data (500 MHz, DMSO-*d*_6_, *δ* in ppm, *J* in Hz) and ^13^C NMR data (125 MHz, DMSO-*d*_6_, *δ* in ppm) for **1**.

Position	*δ*_H_ (*J* in Hz)	*δ*_C_, type
1		177.2, C
2	4.46, m	68.4, CH
3	2.57, dd (17.5, 9.0) 1.58, dd (13.0, 9.0)	37.9, CH_2_
4		84.8, C
5	3.95, t (6.0)	75.5, CH
6	5.59, dd (15.0, 6.0)	129.6, CH
7	6.24, dd (15.0, 10.5)	132.6, CH
8	6.04, dd (15.0, 10.5)	127.5, CH
9	5.64, dd (15.0, 7.5)	140.4, CH
10	2.39, m	34.2, CH
11	2.06, m 1.95, dd (13.0, 7.5)	46.9, CH_2_
12		134.1, C
13	5.74, d (11.0)	126.4, CH
14	6.17, dd (15.0, 11.0)	124.8, CH
15	5.43, dd (15.0, 8.0)	138.0, CH
16	2.06, m	37.9, CH
17	1.29, m	29.3, CH_2_
18	0.82, t (7.0)	11.7, CH_3_
19	1.24, s	24.5, CH_3_
20	0.92, d (6.5)	19.8, CH_3_
21	1.67, s	16.3, CH_3_
22	0.95, d (7.0)	20.1, CH_3_
2-OH	5.82, d (6.5)	
4-OH	5.50, d (5.0)	

Trichodermamide G (**3**): yellow, amorphous powder; [*α*]^20^_D_ −116.5 (*c* 0.50, MeOH); UV (MeOH) *λ*_max_ (log *ε*) 204 (4.40), 274 (3.96), 324 (4.28) nm; ECD (1.55 mM, MeOH) *λ*_max_ (Δ*ε*) 226 (−3.08), 243 (+1.09), 325 (−5.07) nm; ^1^H and ^13^C NMR data, see Table 2; HRESIMS *m/z* 449.1026 [M +H]^+^ (calculated for C_20_H_21_O_8_N_2_S, 449.1013).

**TABLE 2:** ^1^H NMR data (500 MHz, DMSO-*d*_6_, *δ* in ppm, *J* in Hz) and ^13^C NMR data (125 MHz, DMSO-*d*_6_, *δ* in ppm) for **3**.

Position	*δ*_H_ (*J* in Hz)	*δ*_C_, type
1		162.7, C
2	5.18, s	58.9, CH
3	3.29, s	44.6, CH
4		70.3, C
5	6.04, d (8.0)	132.9, CH
6	6.50, t (8.0)	134.7, CH
7	3.42, m	37.8, CH
8	4.02, brs	,72.1, CH
9	3.63, brs	87.0, CH
1′		155.8, C
2′		121.8, C
3′	6.77, s	112.7, C
4′		114.6, C
5′	7.04, d (9.0)	126.7, CH,
6′	6.61, d (9.0)	104.4, CH
7′		153.5, C
8′		136.5, C
9′		147.6, C
7′-OMe	3.81, s	55.9, CH_3_
8′-OMe	3.69, s	60.3, CH_3_
1-NH	10.09, s	

### Antifungal Assays

Antifungal activities were evaluated using the conventional broth dilution assay (Appendino et al., 2008). Five phytopathogenic fungal strains, including *Botrytis cinerea*, Magnaporthe *grisea*, *Phytophthora parasitica*, *Pestallozzia theae*, and *Valsa mali* were used. Carbendazim was used as a positive control.

## Results and Discussion

### Structural Elucidation of the Isolated Compounds

**FIGURE 2:**
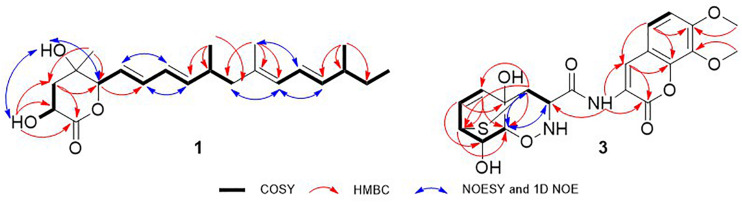
COSY, key HMBC, 1D NOE, and NOESY correlations of **1** and **3**.

Nafuredin C (**1**) was isolated as a white, amorphous powder. Its molecular formula, C_22_H_34_O_4_, was established from high-resolution ESIMS (HRESIMS), ^1^H and ^13^C nuclear magnetic resonance (NMR) data (Supplementary Figures S1, S2, S7), indicating six indexes of hydrogen deficiency. The ^1^H NMR (Table 1) spectrum displayed seven olefinic protons at *δ*_H_ 6.24 (1H, dd, *J* = 15.0, 10.5 Hz), 6.17 (1H, dd, *J* = 15.0, 11.0 Hz), 6.04 (1H, dd, *J* = 15.0, 10.5 Hz), 5.74 (1H, d, *J* = 11.0 Hz), 5.64 (1H, dd, *J* = 15.0, 7.5 Hz), 5.59 (1H, dd, *J* = 15.0, 6.0 Hz), and 5.43 (1H, dd, *J* = 15.0, 8.0 Hz), two hydroxyl signals at *δ*_H_ 5.82 (1H, d, *J* = 6.5 Hz) and 5.50 (1H, d, *J* = 5.0 Hz), two oxygenated methines at *δ*_H_ 4.46 (1H, m) and 3.95 (1H, t, *J* = 6.0 Hz), and five methyls at *δ*_H_ 1.67 (3H, s), 1.24 (3H, s), 0.95 (3H, d, *J* = 7.0 Hz), 0.92 (3H, d, *J* = 6.5 Hz), and 0.82 (3H, t, *J* = 7.0 Hz). The ^13^C NMR and distortionless enhancement by polarization transfer (DEPT) spectra revealed 22 carbon signals, including one ester carbonyl group (*δ*_C_ 177.2), two quaternary carbons (one oxygenated, one olefinic), three methylene groups, 11 methines (seven olefinic, two oxygenated), and five methyl groups. These spectroscopic data are similar to those of nafuredin B, isolated from a mixed culture of the deep-sea-derived fungus *Talaromyces aculeatus* and mangrove-derived fungus *Penicillium variabile* (Zhang et al., 2017). The major differences were replacement of the *sp*^2^ double bond at C-2 and C-3 in nafuredin B by a hydroxyl group at C-2 in **1**, which were consistent with the downfield shifts of C-1/C-4 (*δ*_C_ 163.3/68.0 in nafuredin B *vs* 177.2/84.8 in **1**) and upfield shifts of C-2/C-3 (*δ*_C_ 118.0/156.3 in nafuredin B *vs* 68.4/37.9 in **1**), supported by the COSY correlations of H-2/H-3, and key HMBC from H-2 to C-1/C-4, from H-3 to C-1/C-5, from H-5 to C-3/C-4, from 2-OH to C-1/C-2/C-3, and from H_3_-19 to C-3/C-4/C-5 (Figure 2 and Supplementary Figures S4, S5).

**FIGURE 3:**
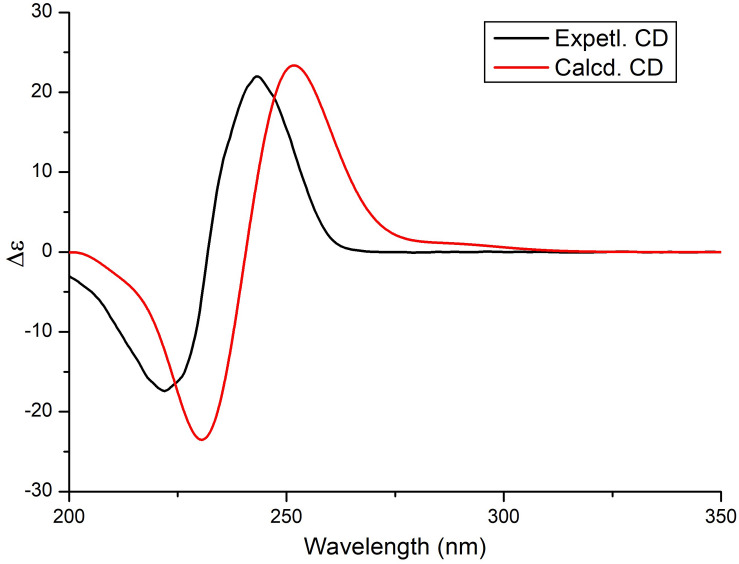
Experimental and theoretical ECD spectra of compound **1**.

The relative configuration of the *δ*-lactone ring was established based on the NOESY spectrum (Figure 2 and Supplementary Figure S6). The correlations of 4-OH with 2-OH and H-5 and of H-6 with H_3_-19 indicated a cofacial relationship among 4-OH, 2-OH, and H-5. The geometry of double bonds in the olefinic chain was *E* elucidated from the *J* values and NOESY correlations (Table 1 and Figure 2). Considering the biosynthesis and coisolation of nafuredin A (**2**), whose absolute configuration was determined based on asymmetric synthesis (Takano et al., 2001; Zhang et al., 2017), the chirality of C-10 and C-16 was determined to be 10*R* and 16*S*, which was supported by the identical NMR data. Comparing the computed ECD spectra with experimental results is a valid method of assigning the absolute configurations of natural products (Cao et al., 2020). To determine the absolute configuration of the *δ*-lactone ring in **1**, ECD computations for all B3LYP/6-311+G(d)-optimized conformers (50 structures) were carried out at the B3LYP/6-311++G(2d,p) level, and six structures with relative energy less than 2.5 kcal/mol were obtained. Boltzmann statistics were performed for ECD simulations with a standard deviation of σ 0.25 eV. The experimental and calculated ECD spectra for the (2*R*,4*S*,5*R*,10*R*,16*S*)-**1** showed good agreement (Figure 3 and Supplementary Table S1), thus suggesting the absolute configuration.

Trichodermamide C (**3**) was obtained as a yellow, amorphous powder, with the molecular formula C_20_H_20_N_2_O_8_S based on its HRESIMS, ^1^H and ^13^C NMR data, indicating 12 degrees of unsaturation (Supplementary Figures S8, S9, S15). The ^13^C NMR and DEPT spectroscopic data (Table 2) revealed 20 carbon signals containing two carbonyl groups, two methoxyl groups, 10 methines (with five olefinic or aromatic, two thiogenated, one nitrogenated, two oxygenated), and six quaternary carbons (with five *sp*^2^ and one *sp*^3^). In examining the ^1^H NMR and HSQC data (Supplementary Figures S8, S10), an amide proton at *δ*_H_ 10.09, two *ortho*-aromatic protons attributed to a 1,2,3,4-tetrasubstituted phenyl unit at *δ*_H_ 7.04 (1H, d, *J* = 9.0 Hz) and 6.61 (1H, d, *J* = 9.0 Hz), and three olefinic protons including two derived from a *cis*-coupled double bond at *δ*_H_ 6.50 (1H, t, *J* = 8.0 Hz) and 6.04 (1H, d, *J* = 8.0 Hz) were observed in the deshielded region of the spectrum, whereas shielded signals for five methines at *δ*_H_ 5.18 (1H, s), 4.02 (1H, s), 3.63 (1H, s), 3.42 (1H, d, *J* = 9.5 Hz), and 3.29 (1H, s) were also present. Additionally, two *O*-methyl groups (*δ*_H_ 3.81 and 3.69) were observed in the ^1^H NMR spectrum of **3**. Inspection of the above NMR data indicated that the isocoumarin portion of **3** was similar to that of aspergillazine A, a modified dipeptide isolated from the marine-derived fungus *Spicaria elegans* (Liu et al., 2005), whereas the signals of oxazine were obviously changed, possibly because of the different location of the sulfur bridge. The characteristic NMR data of C-3 (*δ*_H_ 3.29, *δ*_C_ 44.6) and C-7 (*δ*_H_ 3.42, *δ*_C_ 37.8) indicated they were linked by a sulfur atom, which was confirmed from the COSY correlation of H5/H6/H7/H8/H9 and HMBC correlations from H-3 to C-5/C-7/C-9, from H-5 to C-7/C-9, from H-6 to C-4/C-8, and from H-7 to C-9 (Figure 2 and Supplementary Figures S11, S12). In the NOE difference spectra (Figure 2 and Supplementary Figures S13, S14), irradiation of H-2 and H-3 enhanced the resonance of H-9. This information, in addition to the similar coupling constant of H8/H-9 (brs) to trichodermamide A and aspergillazine A (Capon et al., 2005; Liu et al., 2005), indicates a *syn* relationship among H-2, H-3, H-9, and 8-OH. 4-OH was assigned on the opposite face of the sulfur bridge, as the *cis* relationship did not lead to a reasonable model according to 3D simulations.

**FIGURE 4:**
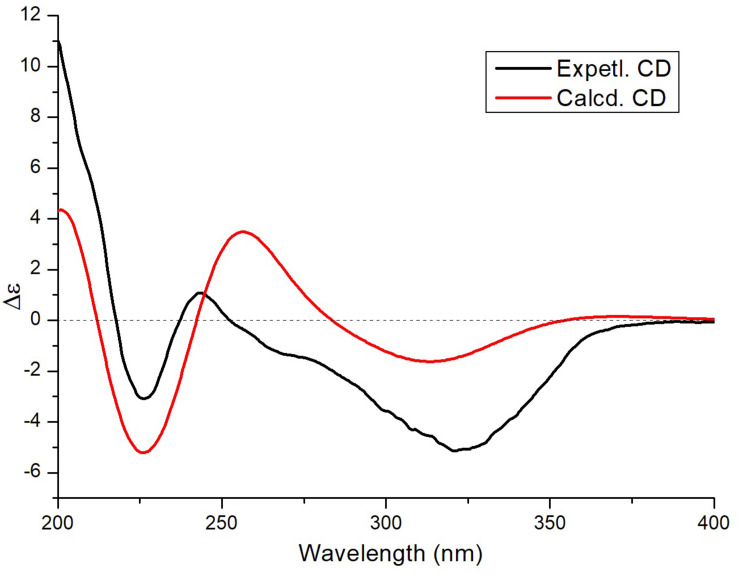
Experimental and theoretical ECD spectra of compound **3**.

To determine the absolute configuration of **3**, ECD calculations were carried out. Monte Carlo conformational searches were applied using Spartan’s 10 software with the Merck molecular force field. Conformers with a Boltzmann-population of over 10% were selected for ECD calculations and were initially optimized at the B3LYP/6-31g (d,p) level in MeOH using the conductor-like polarizable continuum calculation model. Theoretical calculation of the ECD was conducted in MeOH using time-dependent density functional theory at the B3LYP/6-31+g (d,p) level for all conformers of compound (2*R*,3*S*,4*S*,7*S*,8*S*,9*S*)-**3**. Rotatory strengths for a total of 30 excited states were calculated. ECD spectra were generated using the programs SpecDis 1.6 (University of Würzburg, Würzburg, Germany) and GraphPad Prism 5 (GraphPad, Inc., La Jolla, CA, United States) from dipole-length rotational strengths by applying Gaussian band shapes with sigma = 0.3 eV. The predicted ECD spectrum agreed well with the experimental result (Figure 4 and Supplementary Tables S2, S3), indicating that the absolute configuration of **3** was 2*R*,3*S*,4*S*,7*S*,8*S*,9*S*.

Epipolythiodiketopiperazine alkaloids are fungal metabolites with a highly complex molecular architecture comprising a densely functionalized core structure with many stereogenic centers. In the past decade, an increasing number of studies have discovered powerful new biological processes involving these molecules, including cytotoxic, antileukemic, antiviral, antibiotic, and antinematodal activities (Kim and Movassaghi, 2015); however, epithiodiketopiperazines have not been widely examined. Notably, trichodermamide G is a novel epithiodiketopiperazine derivative with an unprecedented cyclic system containing a sulfur bridge.

Compounds **2** and **4**–**6** were identified as nafuredin A (Ui et al., 2001), trichodermamide A (Liu et al., 2005), aspergillazine A (Liu et al., 2005), and peniisocoumarin H (Cai et al., 2018) by comparison of their spectroscopic data with those in the literature.

### Antifungal Activity

In the present study, all isolated compounds were evaluated to determine their anti-phytopathogenic fungal activities against *B. cinerea*, *M. oryzae*, *P. theae*, *P. parasitica*, and *V. mali*. The nafuredin derivatives **1** and **2** exhibited obvious antifungal activities against *M. oryzae*, with minimum inhibitory concentrations (MICs) of 8.63 and 17.4 μM, respectively. These data indicate that the antifungal activity toward *M. oryzae* of new compound **1** was the same magnitude as that of the positive control carbendazim (MIC = 3.27 μM). Compounds **1** and **2** also displayed weak antifungal activity against *V. mali* and *P. theae* compared to carbendazim (Table 3).

**TABLE 3:** Antifungal activity of **1** and **2**.

Compounds	MIC (*μ*M)		
	*M. oryzae*	*P. theae*	*V. mali*
**1**	8.63	553	34.5
**2**	17.4	-	16.7
Carbendazim	3.27	0.82	0.82

*“–” means no antifungal activity.*

## Conclusion

In summary, we report three polyketide derivatives, nafuredin C (**1**), nafuredin A (**2**), and peniisocoumarin H (**6**), and three epithiodiketopiperazine derivatives, trichodermamide G (**3**), trichodermamide A (**4**), and aspergillazin A (**5**), isolated from the mangrove-derived fungus *T. harzianum* D13. Among them, nafuredin C (**1**) and trichodermamide G (**3**) are new compounds. Their structures were assigned based on extensive NMR spectroscopic data, time-dependent density functional theory ECD calculations together with comparison of their ECD spectra. Trichodermamide G contains a unique sulfur bridge compared to the homologous compounds. The polyketide derivatives nafuredin C (**1**) and nafuredin A (**2**) exhibited distinct antifungal activity against *M. oryzae*. *T. harzianum* have been widely used as biocontrol agents and commercially marketed as biopesticides. Our study expands the source of *Trichoderma* which used for biocontrol, and provides basic material for the discovery of new antifungal pesticides.

## Data Availability Statement

The raw data supporting the conclusions of this article will be made available by the authors, without undue reservation, to any qualified researcher.

## Author Contributions

D-LZ and C-SZ conceived and designed the experiments. X-FZ R-HH, DW, X-QW, and PZ performed the experiments. Y-QL, C-JZ, and PZ analyzed the data. D-LZ wrote the manuscript. All authors reviewed the manuscript.

## Conflict of Interest

The authors declare that the research was conducted in the absence of any commercial or financial relationships that could be construed as a potential conflict of interest.
