# Supplementary material for: Antifungal Nafuredin and Epithiodiketopiperazine Derivatives From the Mangrove-Derived Fungus Trichoderma harzianum D13
Source: Front Microbiol. 2020 Jun 26;11:1495. doi: 10.3389/fmicb.2020.01495 (PMC7333244; doi:10.3389/fmicb.2020.01495)
Supplement: Supplementary file 1 [file Data_Sheet_1.docx]

***Supporting Information***

Antifungal Nafuredin and Epithiodiketopiperazine Derivatives from the Mangrove-Derived Fungus *Trichoderma harzianum* D13

Dong-Lin Zhao^1†^, Xi-Fen Zhang^1†^, Rui-Huan Huang^1^, Dan Wang^1,3^, Xiao-Qiang Wang^1^, Yi-Qiang Li^1^, Cai-Juan Zheng^2^, Peng Zhang^1^*, and Cheng-Sheng Zhang^1^*

^1^Tobacco Research Institute of Chinese Academy of Agricultural Sciences, Qingdao 266101, People’s Republic of China

^2^Key Laboratory of Tropical Medicinal Resource Chemistry of Ministry of Education, Hainan Normal University, Haikou 571158, People’s Republic of China

^3^Plant Protection Station of Shandong Province, Jinan 250100, People’s Republic of China

***Correspondence:**Peng Zhang
zhangpeng@caas.cn

Chengsheng Zhang
zhchengsheng@126.com

^†^These authors contributed equally to this work.

**Keywords: Nafuredins, Epithiodiketopiperazines, *Magnaporthe oryzae*, Antifungal activity, *Trichoderma harzianum***

**List of Supporting Information**

**Table S1.** The coordinate for the lowest-energy conformer **1** in ECD calculation

**Table S2.** Gibbs free energies and equilibrium populations of low-energy conformers of **3**

**Table S3.** Cartesian coordinates for the low-energy reoptimized MMFF conformers of **3** at B3LYP/6-31+G(d,p) level of theory in CH_3_OH

**Figure S1.** ^1^H NMR (500 MHz, DMSO-*d*_6_) spectrum of compound **1**

**Figure S2.** ^13^C NMR (125 MHz, DMSO-*d*_6_) spectrum of compound **1**

**Figure S3.** HMQC (DMSO-*d*_6_) spectrum of compound **1**

**Figure S4.** COSY (DMSO-*d*_6_) spectrum of compound **1**

**Figure S5.** HMBC (DMSO-*d*_6_) spectrum of compound **1**

**Figure S6.** NOESY (DMSO-*d*_6_) spectrum of compound **1**

**Figure S7.** HRESIMS spectrum of compound **1**

**Figure S8.** ^1^H NMR (500 MHz, DMSO-*d*_6_) spectrum of compound **3**

**Figure S9.** ^13^C NMR (125 MHz, DMSO-*d*_6_) spectrum of compound **3**

**Figure S10.** HMQC (DMSO-*d*_6_) spectrum of compound **3**

**Figure S11.** COSY (DMSO-*d*_6_) spectrum of compound **3**

**Figure S12.** HMBC (DMSO-*d*_6_) spectrum of compound **3**

**Figure S13.** 1D NOE (DMSO-*d*_6_) spectrum of compound **3**

**Figure S14.** 1D NOE (DMSO-*d*_6_) spectrum of compound **3**

**Figure S15.** HRESIMS spectrum of compound **3**

**Table S1.** The coordinate for the lowest-energy conformer **1** in ECD calculation.

----------------------------------------------------------------------------------

Coordinates (Angstroms)

X Y Z

-----------------------------------------------------------------------------------

C -5.47663100 0.48279700 -1.49027800

C -5.60564000 1.75091100 -0.66082400

C -5.27744800 1.58882300 0.83264000

O -5.14321700 0.36735000 1.36393900

C -5.14146900 -0.86567200 0.58869200

C -5.98046700 -0.73742600 -0.71560600

O -5.21854600 2.56663100 1.53899500

O -6.97671900 2.18266600 -0.73954700

C -3.72921300 -1.34755400 0.41049700

C -2.61549300 -0.68261500 0.75035000

C -1.26831700 -1.20146100 0.57502600

C -0.15591000 -0.52846900 0.90442600

C 1.25266000 -1.02369500 0.73229000

C 1.99373100 -0.14729900 -0.32010900

C 1.99392000 -1.03449000 2.07938200

C 3.40040100 -0.58974500 -0.65605600

C 4.44768300 0.21966200 -0.39865000

C 3.51915000 -1.94719800 -1.30108800

C 5.85603600 -0.02489400 -0.66809400

C 6.83408800 0.85275000 -0.39435300

C 8.29914800 0.65765800 -0.67549600

C 9.14541200 0.73365700 0.61527200

C 8.85434100 -0.37696600 1.62592400

C 8.79034800 1.69439200 -1.70337900

C -5.90030600 -2.01366300 -1.55191400

O -7.35384200 -0.59233400 -0.34040600

H -4.42641700 0.33126400 -1.75413400

H -6.03584300 0.60930900 -2.42155300

H -4.95337600 2.54194000 -1.04420700

H -5.68625400 -1.56359200 1.23146600

H -7.09305100 2.94784000 -0.15910700

H -3.64215200 -2.35062600 -0.00035800

H -2.69908600 0.30185300 1.20450400

H -1.17740700 -2.20076500 0.14852100

H -0.25803000 0.47031600 1.33316600

H 1.20481500 -2.05102400 0.35228400

H 1.38940400 -0.14890000 -1.23699000

H 2.00974800 0.88838600 0.03743500

H 3.02075600 -1.39025200 1.96392700

H 1.48591000 -1.67780400 2.80219800

H 2.04344700 -0.02744500 2.50707200

H 4.23187300 1.18264600 0.06376200

H 2.81546900 -2.04074000 -2.13654600

H 4.51690100 -2.15300000 -1.68740200

H 3.27091700 -2.75052500 -0.59747000

H 6.13418600 -0.97576900 -1.11776800

H 6.56674800 1.80936200 0.06048100

H 8.43506800 -0.34209200 -1.11001400

H 10.20542000 0.70319600 0.33576400

H 8.98788400 1.71329200 1.08551900

H 9.04698400 -1.36526400 1.19542800

H 9.48514300 -0.27701700 2.51388700

H 7.81163300 -0.36085800 1.95346500

H 9.85060700 1.54805100 -1.93221700

H 8.22843300 1.62313100 -2.63806900

H 8.67196400 2.71432900 -1.32227800

H -4.89938100 -2.19267200 -1.94891700

H -6.20545200 -2.88121200 -0.96067300

H -6.59577900 -1.92568100 -2.38839200

H -7.61862000 0.33877800 -0.38855000

H 0.70694300 2.28702900 -0.38699400

-----------------------------------------------------------------------------------

**Table S2.** Gibbs free energies*^a^* and equilibrium populations*^b^* of low-energy conformers of **3**.

| Conformers | In MeOH | |
| --- | --- | --- |
|  | *G* | *P* (%) |
| **3-1** | -1181751.9763257 | 18.35 |
| **3-2** | -1181751.88784679 | 15.80 |
| **3-3** | -1181752.12504557 | 23.59 |
| **3-4** | -1181752.13634075 | 24.04 |
| **3-5** | -1181751.97193313 | 18.21 |
| *^a^*B3LYP/6-31+G(d,p), in kcal/mol. *^b^*From *G* values at 298.15K. | | |

**Table S3.** Cartesian coordinates for the low-energy reoptimized MMFF conformers of **3** at B3LYP/6-31+G(d,p) level of theory in CH_3_OH.

| **3-1** | | Standard Orientation  (Ångstroms) | | | |
| --- | --- | --- | --- | --- | --- |
| Center number | Atomic number | Atomic Type | X | Y | Z |
| 1. | 6. | 0. | 5.540462 | 0.661167 | 1.888764 |
| 2. | 6. | 0. | 4.361739 | 1.416623 | 1.338704 |
| 3. | 6. | 0. | 4.536404 | 1.680837 | -0.170521 |
| 4. | 6. | 0. | 4.942070 | 0.358286 | -0.875617 |
| 5. | 6. | 0. | 4.946537 | -0.843299 | 0.105799 |
| 6. | 6. | 0. | 5.822034 | -0.490569 | 1.272504 |
| 7. | 8. | 0. | 4.152557 | 0.004720 | -2.020035 |
| 8. | 7. | 0. | 2.754782 | -0.110303 | -1.629627 |
| 9. | 6. | 0. | 2.597029 | -1.276173 | -0.748323 |
| 10. | 6. | 0. | 3.445452 | -1.067483 | 0.533653 |
| 11. | 16. | 0. | 2.845741 | 0.351227 | 1.564721 |
| 12. | 1. | 0. | 5.951346 | 0.470549 | -1.280617 |
| 13. | 8. | 0. | 5.546556 | 2.678046 | -0.302923 |
| 14. | 6. | 0. | 1.117957 | -1.524828 | -0.424089 |
| 15. | 8. | 0. | 0.743004 | -2.628467 | -0.049008 |
| 16. | 7. | 0. | 0.304407 | -0.438978 | -0.594561 |
| 17. | 6. | 0. | -1.073738 | -0.374094 | -0.383117 |
| 18. | 6. | 0. | -1.609068 | 0.964538 | -0.671978 |
| 19. | 8. | 0. | -2.960924 | 1.141933 | -0.518463 |
| 20. | 6. | 0. | -3.798241 | 0.135191 | -0.108891 |
| 21. | 6. | 0. | -3.299371 | -1.146197 | 0.183504 |
| 22. | 6. | 0. | -1.889572 | -1.382501 | 0.030396 |
| 23. | 6. | 0. | -5.157974 | 0.455552 | 0.014182 |
| 24. | 6. | 0. | -6.045603 | -0.553327 | 0.424568 |
| 25. | 6. | 0. | -5.561738 | -1.838692 | 0.720165 |
| 26. | 6. | 0. | -4.212579 | -2.130167 | 0.608987 |
| 27. | 8. | 0. | -7.377317 | -0.308067 | 0.613467 |
| 28. | 6. | 0. | -8.131516 | 0.080327 | -0.544064 |
| 29. | 8. | 0. | -5.624918 | 1.704667 | -0.300716 |
| 30. | 6. | 0. | -5.343014 | 2.725435 | 0.668384 |
| 31. | 8. | 0. | -0.930215 | 1.904796 | -1.032184 |
| 32. | 8. | 0. | 5.438648 | -2.005014 | -0.538281 |
| 33. | 1. | 0. | 6.082345 | 1.030396 | 2.752943 |
| 34. | 1. | 0. | 4.159192 | 2.353310 | 1.859364 |
| 35. | 1. | 0. | 3.582803 | 2.023429 | -0.586098 |
| 36. | 1. | 0. | 6.623070 | -1.168320 | 1.545914 |
| 37. | 1. | 0. | 2.321499 | -0.305819 | -2.531145 |
| 38. | 1. | 0. | 2.950064 | -2.208430 | -1.211306 |
| 39. | 1. | 0. | 3.380893 | -1.977357 | 1.133539 |
| 40. | 1. | 0. | 5.515532 | 3.009151 | -1.210360 |
| 41. | 1. | 0. | 0.744732 | 0.451439 | -0.817647 |
| 42. | 1. | 0. | -1.473434 | -2.358537 | 0.243847 |
| 43. | 1. | 0. | -6.276528 | -2.587027 | 1.044399 |
| 44. | 1. | 0. | -3.844340 | -3.124676 | 0.840513 |
| 45. | 1. | 0. | -9.159920 | 0.204635 | -0.200597 |
| 46. | 1. | 0. | -7.762468 | 1.017679 | -0.965610 |
| 47. | 1. | 0. | -8.097964 | -0.707545 | -1.306573 |
| 48. | 1. | 0. | -5.824337 | 3.632095 | 0.298362 |
| 49. | 1. | 0. | -4.266425 | 2.897494 | 0.758571 |
| 50. | 1. | 0. | -5.763243 | 2.460172 | 1.645125 |
| 51. | 1. | 0. | 5.188255 | -1.931975 | -1.471763 |
| **3-2** | | Standard Orientation  (Ångstroms) | | | |
| Center number | Atom number | Type | X | Y | Z |
| 1. | 6. | 0. | 5.464588 | 0.318129 | 2.084089 |
| 2. | 6. | 0. | 4.306316 | 1.166307 | 1.636072 |
| 3. | 6. | 0. | 4.539300 | 1.695456 | 0.206527 |
| 4. | 6. | 0. | 4.965546 | 0.516932 | -0.710514 |
| 5. | 6. | 0. | 4.937745 | -0.840542 | 0.040368 |
| 6. | 6. | 0. | 5.770663 | -0.705525 | 1.281662 |
| 7. | 8. | 0. | 4.212347 | 0.380199 | -1.924350 |
| 8. | 7. | 0. | 2.802026 | 0.202928 | -1.607043 |
| 9. | 6. | 0. | 2.619577 | -1.102119 | -0.956931 |
| 10. | 6. | 0. | 3.423530 | -1.129208 | 0.369733 |
| 11. | 16. | 0. | 2.783673 | 0.087100 | 1.612408 |
| 12. | 1. | 0. | 5.987054 | 0.692598 | -1.058329 |
| 13. | 8. | 0. | 5.558747 | 2.688195 | 0.294393 |
| 14. | 6. | 0. | 1.134174 | -1.406934 | -0.723665 |
| 15. | 8. | 0. | 0.761615 | -2.560410 | -0.549924 |
| 16. | 7. | 0. | 0.313611 | -0.313081 | -0.726711 |
| 17. | 6. | 0. | -1.065176 | -0.293978 | -0.507700 |
| 18. | 6. | 0. | -1.610581 | 1.070274 | -0.563867 |
| 19. | 8. | 0. | -2.959131 | 1.212006 | -0.352575 |
| 20. | 6. | 0. | -3.786678 | 0.144967 | -0.110018 |
| 21. | 6. | 0. | -3.282969 | -1.166848 | -0.071988 |
| 22. | 6. | 0. | -1.873703 | -1.364438 | -0.275472 |
| 23. | 6. | 0. | -5.146532 | 0.430127 | 0.080048 |
| 24. | 6. | 0. | -6.022177 | -0.638127 | 0.336729 |
| 25. | 6. | 0. | -5.533136 | -1.954414 | 0.378271 |
| 26. | 6. | 0. | -4.189268 | -2.216899 | 0.171320 |
| 27. | 8. | 0. | -7.367712 | -0.445305 | 0.484630 |
| 28. | 6. | 0. | -7.784121 | 0.344184 | 1.608544 |
| 29. | 8. | 0. | -5.609859 | 1.719666 | 0.064483 |
| 30. | 6. | 0. | -5.726027 | 2.310445 | -1.238182 |
| 31. | 8. | 0. | -0.943201 | 2.060251 | -0.785630 |
| 32. | 8. | 0. | 5.453486 | -1.871358 | -0.782702 |
| 33. | 1. | 0. | 5.974806 | 0.524096 | 3.018983 |
| 34. | 1. | 0. | 4.084388 | 1.995970 | 2.308220 |
| 35. | 1. | 0. | 3.604629 | 2.118708 | -0.176067 |
| 36. | 1. | 0. | 6.562982 | -1.424570 | 1.457653 |
| 37. | 1. | 0. | 2.400425 | 0.173885 | -2.543346 |
| 38. | 1. | 0. | 2.990946 | -1.936146 | -1.569101 |
| 39. | 1. | 0. | 3.338996 | -2.130679 | 0.795904 |
| 40. | 1. | 0. | 5.565168 | 3.177743 | -0.538893 |
| 41. | 1. | 0. | 0.748317 | 0.605393 | -0.787786 |
| 42. | 1. | 0. | -1.451912 | -2.360489 | -0.240392 |
| 43. | 1. | 0. | -6.241858 | -2.752703 | 0.569388 |
| 44. | 1. | 0. | -3.816731 | -3.235921 | 0.204488 |
| 45. | 1. | 0. | -8.874308 | 0.370453 | 1.565874 |
| 46. | 1. | 0. | -7.470348 | -0.128463 | 2.547363 |
| 47. | 1. | 0. | -7.383571 | 1.358712 | 1.554176 |
| 48. | 1. | 0. | -6.150828 | 3.303238 | -1.081542 |
| 49. | 1. | 0. | -6.396881 | 1.721090 | -1.873573 |
| 50. | 1. | 0. | -4.746619 | 2.406784 | -1.716298 |
| 51. | 1. | 0. | 5.230341 | -1.634927 | -1.695737 |

| **3-3** | | Standard Orientation  (Ångstroms) | | | |
| --- | --- | --- | --- | --- | --- |
| Center number | Atom number | Type | X | Y | Z |
| 1. | 6. | 0. | 5.546091 | 0.669504 | 1.880668 |
| 2. | 6. | 0. | 4.366990 | 1.420513 | 1.327352 |
| 3. | 6. | 0. | 4.542218 | 1.674542 | -0.192423 |
| 4. | 6. | 0. | 4.941945 | 0.354171 | -0.885780 |
| 5. | 6. | 0. | 4.947744 | -0.840427 | 0.104354 |
| 6. | 6. | 0. | 5.826349 | -0.483933 | 1.267096 |
| 7. | 8. | 0. | 4.147110 | -0.007589 | -2.021038 |
| 8. | 7. | 0. | 2.751585 | -0.118485 | -1.626402 |
| 9. | 6. | 0. | 2.594889 | -1.279830 | -0.739580 |
| 10. | 6. | 0. | 3.447043 | -1.063588 | 0.538612 |
| 11. | 16. | 0. | 2.847648 | 0.359485 | 1.564109 |
| 12. | 1. | 0. | 5.946455 | 0.472619 | -1.298832 |
| 13. | 8. | 0. | 5.591413 | 2.604900 | -0.445000 |
| 14. | 6. | 0. | 1.116681 | -1.526328 | -0.410748 |
| 15. | 8. | 0. | 0.740578 | -2.628111 | -0.031385 |
| 16. | 7. | 0. | 0.304042 | -0.439710 | -0.583006 |
| 17. | 6. | 0. | -1.073932 | -0.373232 | -0.372272 |
| 18. | 6. | 0. | -1.607658 | 0.966712 | -0.658045 |
| 19. | 8. | 0. | -2.960237 | 1.143904 | -0.509535 |
| 20. | 6. | 0. | -3.799214 | 0.136568 | -0.104901 |
| 21. | 6. | 0. | -3.301337 | -1.144934 | 0.188593 |
| 22. | 6. | 0. | -1.891251 | -1.381535 | 0.038782 |
| 23. | 6. | 0. | -5.159663 | 0.456229 | 0.012009 |
| 24. | 6. | 0. | -6.048632 | -0.552904 | 0.418562 |
| 25. | 6. | 0. | -5.565698 | -1.838155 | 0.716070 |
| 26. | 6. | 0. | -4.216028 | -2.129273 | 0.610093 |
| 27. | 8. | 0. | -7.381245 | -0.307441 | 0.601559 |
| 28. | 6. | 0. | -8.130412 | 0.076127 | -0.560838 |
| 29. | 8. | 0. | -5.626109 | 1.704874 | -0.305778 |
| 30. | 6. | 0. | -5.349860 | 2.726392 | 0.663988 |
| 31. | 8. | 0. | -0.927260 | 1.908504 | -1.011330 |
| 32. | 8. | 0. | 5.435577 | -2.006114 | -0.534891 |
| 33. | 1. | 0. | 6.090457 | 1.040077 | 2.742975 |
| 34. | 1. | 0. | 4.158375 | 2.354461 | 1.854711 |
| 35. | 1. | 0. | 3.590862 | 2.019352 | -0.611552 |
| 36. | 1. | 0. | 6.627966 | -1.160757 | 1.541049 |
| 37. | 1. | 0. | 2.315098 | -0.315514 | -2.525966 |
| 38. | 1. | 0. | 2.947361 | -2.214596 | -1.197835 |
| 39. | 1. | 0. | 3.385734 | -1.969736 | 1.144414 |
| 40. | 1. | 0. | 5.270706 | 3.486526 | -0.213112 |
| 41. | 1. | 0. | 0.745819 | 0.449074 | -0.809825 |
| 42. | 1. | 0. | -1.475941 | -2.357985 | 0.251962 |
| 43. | 1. | 0. | -6.281586 | -2.586768 | 1.037237 |
| 44. | 1. | 0. | -3.848480 | -3.123853 | 0.842416 |
| 45. | 1. | 0. | -9.160494 | 0.201070 | -0.222661 |
| 46. | 1. | 0. | -7.759948 | 1.012192 | -0.984101 |
| 47. | 1. | 0. | -8.092881 | -0.714460 | -1.320359 |
| 48. | 1. | 0. | -5.830331 | 3.632389 | 0.291193 |
| 49. | 1. | 0. | -4.273852 | 2.899555 | 0.759120 |
| 50. | 1. | 0. | -5.774452 | 2.461328 | 1.638903 |
| 51. | 1. | 0. | 5.191172 | -1.932506 | -1.470018 |

| **3-4** | | Standard Orientation  (Ångstroms) | | | |
| --- | --- | --- | --- | --- | --- |
| Center number | Atom number | Type | X | Y | Z |
| 1. | 6. | 0. | 5.442504 | 0.366926 | 2.091016 |
| 2. | 6. | 0. | 4.286711 | 1.200775 | 1.613203 |
| 3. | 6. | 0. | 4.531805 | 1.697437 | 0.164729 |
| 4. | 6. | 0. | 4.965148 | 0.505319 | -0.715294 |
| 5. | 6. | 0. | 4.933934 | -0.833869 | 0.067273 |
| 6. | 6. | 0. | 5.757407 | -0.671594 | 1.311368 |
| 7. | 8. | 0. | 4.220105 | 0.336610 | -1.927523 |
| 8. | 7. | 0. | 2.808774 | 0.164181 | -1.617150 |
| 9. | 6. | 0. | 2.623191 | -1.126510 | -0.940531 |
| 10. | 6. | 0. | 3.417475 | -1.120913 | 0.392489 |
| 11. | 16. | 0. | 2.763049 | 0.119466 | 1.603743 |
| 12. | 1. | 0. | 5.985740 | 0.686213 | -1.060710 |
| 13. | 8. | 0. | 5.590197 | 2.650137 | 0.114781 |
| 14. | 6. | 0. | 1.136748 | -1.427994 | -0.710939 |
| 15. | 8. | 0. | 0.761470 | -2.578397 | -0.523157 |
| 16. | 7. | 0. | 0.317840 | -0.332753 | -0.731094 |
| 17. | 6. | 0. | -1.060450 | -0.307532 | -0.511111 |
| 18. | 6. | 0. | -1.601198 | 1.058425 | -0.572677 |
| 19. | 8. | 0. | -2.949392 | 1.205644 | -0.362597 |
| 20. | 6. | 0. | -3.780205 | 0.142784 | -0.113114 |
| 21. | 6. | 0. | -3.280730 | -1.170377 | -0.067270 |
| 22. | 6. | 0. | -1.872391 | -1.373870 | -0.271572 |
| 23. | 6. | 0. | -5.138964 | 0.433522 | 0.076036 |
| 24. | 6. | 0. | -6.017976 | -0.630084 | 0.340120 |
| 25. | 6. | 0. | -5.533202 | -1.947619 | 0.389960 |
| 26. | 6. | 0. | -4.190323 | -2.215802 | 0.183580 |
| 27. | 8. | 0. | -7.362863 | -0.431708 | 0.487546 |
| 28. | 6. | 0. | -7.775685 | 0.366292 | 1.606692 |
| 29. | 8. | 0. | -5.597666 | 1.724695 | 0.051729 |
| 30. | 6. | 0. | -5.718123 | 2.303505 | -1.255896 |
| 31. | 8. | 0. | -0.930475 | 2.045506 | -0.797292 |
| 32. | 8. | 0. | 5.456045 | -1.881380 | -0.729648 |
| 33. | 1. | 0. | 5.945315 | 0.591373 | 3.025923 |
| 34. | 1. | 0. | 4.049305 | 2.037288 | 2.274777 |
| 35. | 1. | 0. | 3.600924 | 2.112211 | -0.236120 |
| 36. | 1. | 0. | 6.549956 | -1.384946 | 1.508165 |
| 37. | 1. | 0. | 2.413686 | 0.116664 | -2.555382 |
| 38. | 1. | 0. | 3.001452 | -1.973555 | -1.529930 |
| 39. | 1. | 0. | 3.333881 | -2.112670 | 0.840942 |
| 40. | 1. | 0. | 5.259132 | 3.483079 | 0.476110 |
| 41. | 1. | 0. | 0.755660 | 0.583503 | -0.803191 |
| 42. | 1. | 0. | -1.453816 | -2.371071 | -0.231029 |
| 43. | 1. | 0. | -6.244310 | -2.742406 | 0.586737 |
| 44. | 1. | 0. | -3.821167 | -3.235837 | 0.222849 |
| 45. | 1. | 0. | -8.865773 | 0.396871 | 1.564158 |
| 46. | 1. | 0. | -7.463646 | -0.101905 | 2.548333 |
| 47. | 1. | 0. | -7.370962 | 1.378820 | 1.546050 |
| 48. | 1. | 0. | -6.137718 | 3.299670 | -1.106749 |
| 49. | 1. | 0. | -6.394779 | 1.711022 | -1.882149 |
| 50. | 1. | 0. | -4.740770 | 2.390832 | -1.740003 |
| 51. | 1. | 0. | 5.249379 | -1.658396 | -1.650053 |

| **3-5** | | Standard Orientation  (Ångstroms) | | | |
| --- | --- | --- | --- | --- | --- |
| Center number | Atom number | Type | X | Y | Z |
| 1. | 6. | 0. | 5.541026 | 0.660835 | 1.888321 |
| 2. | 6. | 0. | 4.362248 | 1.416417 | 1.338595 |
| 3. | 6. | 0. | 4.536638 | 1.680721 | -0.170674 |
| 4. | 6. | 0. | 4.942009 | 0.358200 | -0.875940 |
| 5. | 6. | 0. | 4.946594 | -0.843483 | 0.105406 |
| 6. | 6. | 0. | 5.822411 | -0.490868 | 1.271900 |
| 7. | 8. | 0. | 4.152197 | 0.004752 | -2.020206 |
| 8. | 7. | 0. | 2.754595 | -0.110169 | -1.629435 |
| 9. | 6. | 0. | 2.596900 | -1.276060 | -0.748196 |
| 10. | 6. | 0. | 3.445604 | -1.067581 | 0.533604 |
| 11. | 16. | 0. | 2.846188 | 0.351114 | 1.564906 |
| 12. | 1. | 0. | 5.951203 | 0.470417 | -1.281137 |
| 13. | 8. | 0. | 5.546910 | 2.677787 | -0.303325 |
| 14. | 6. | 0. | 1.117880 | -1.524613 | -0.423638 |
| 15. | 8. | 0. | 0.742907 | -2.628212 | -0.048490 |
| 16. | 7. | 0. | 0.304349 | -0.438730 | -0.594159 |
| 17. | 6. | 0. | -1.073790 | -0.373760 | -0.382651 |
| 18. | 6. | 0. | -1.609067 | 0.964905 | -0.671339 |
| 19. | 8. | 0. | -2.960993 | 1.142270 | -0.517823 |
| 20. | 6. | 0. | -3.798316 | 0.135430 | -0.108586 |
| 21. | 6. | 0. | -3.299455 | -1.145990 | 0.183699 |
| 22. | 6. | 0. | -1.889634 | -1.382210 | 0.030745 |
| 23. | 6. | 0. | -5.158131 | 0.455620 | 0.014261 |
| 24. | 6. | 0. | -6.045736 | -0.553380 | 0.424300 |
| 25. | 6. | 0. | -5.561867 | -1.838787 | 0.719754 |
| 26. | 6. | 0. | -4.212668 | -2.130133 | 0.608801 |
| 27. | 8. | 0. | -7.377493 | -0.308090 | 0.612981 |
| 28. | 6. | 0. | -8.131453 | 0.079435 | -0.545006 |
| 29. | 8. | 0. | -5.625193 | 1.704706 | -0.300585 |
| 30. | 6. | 0. | -5.343477 | 2.725438 | 0.668667 |
| 31. | 8. | 0. | -0.930252 | 1.905212 | -1.031462 |
| 32. | 8. | 0. | 5.438437 | -2.005210 | -0.538853 |
| 33. | 1. | 0. | 6.083148 | 1.029958 | 2.752397 |
| 34. | 1. | 0. | 4.159931 | 2.353101 | 1.859344 |
| 35. | 1. | 0. | 3.582989 | 2.023463 | -0.586002 |
| 36. | 1. | 0. | 6.623514 | -1.168631 | 1.545076 |
| 37. | 1. | 0. | 2.320964 | -0.305447 | -2.530830 |
| 38. | 1. | 0. | 2.949757 | -2.208315 | -1.211342 |
| 39. | 1. | 0. | 3.381086 | -1.977472 | 1.133463 |
| 40. | 1. | 0. | 5.514471 | 3.010253 | -1.210208 |
| 41. | 1. | 0. | 0.744811 | 0.451715 | -0.816872 |
| 42. | 1. | 0. | -1.473434 | -2.358226 | 0.244198 |
| 43. | 1. | 0. | -6.276672 | -2.587227 | 1.043713 |
| 44. | 1. | 0. | -3.844368 | -3.124656 | 0.840175 |
| 45. | 1. | 0. | -9.160112 | 0.203044 | -0.202059 |
| 46. | 1. | 0. | -7.762885 | 1.016960 | -0.966608 |
| 47. | 1. | 0. | -8.096963 | -0.708587 | -1.307323 |
| 48. | 1. | 0. | -5.824714 | 3.632115 | 0.298586 |
| 49. | 1. | 0. | -4.266906 | 2.897444 | 0.759093 |
| 50. | 1. | 0. | -5.763945 | 2.460089 | 1.645278 |
| 51. | 1. | 0. | 5.188044 | -1.932040 | -1.472319 |


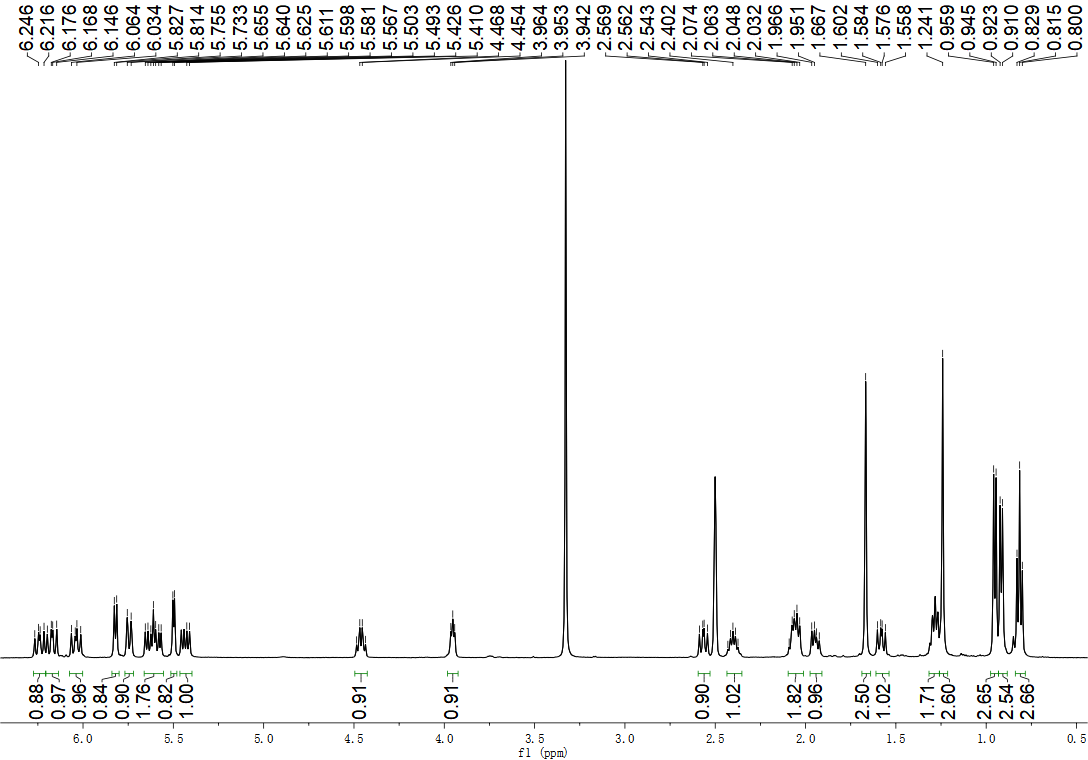


**Figure S1.** ^1^H NMR (500 MHz, DMSO-*d*_6_) spectrum of compound **1**


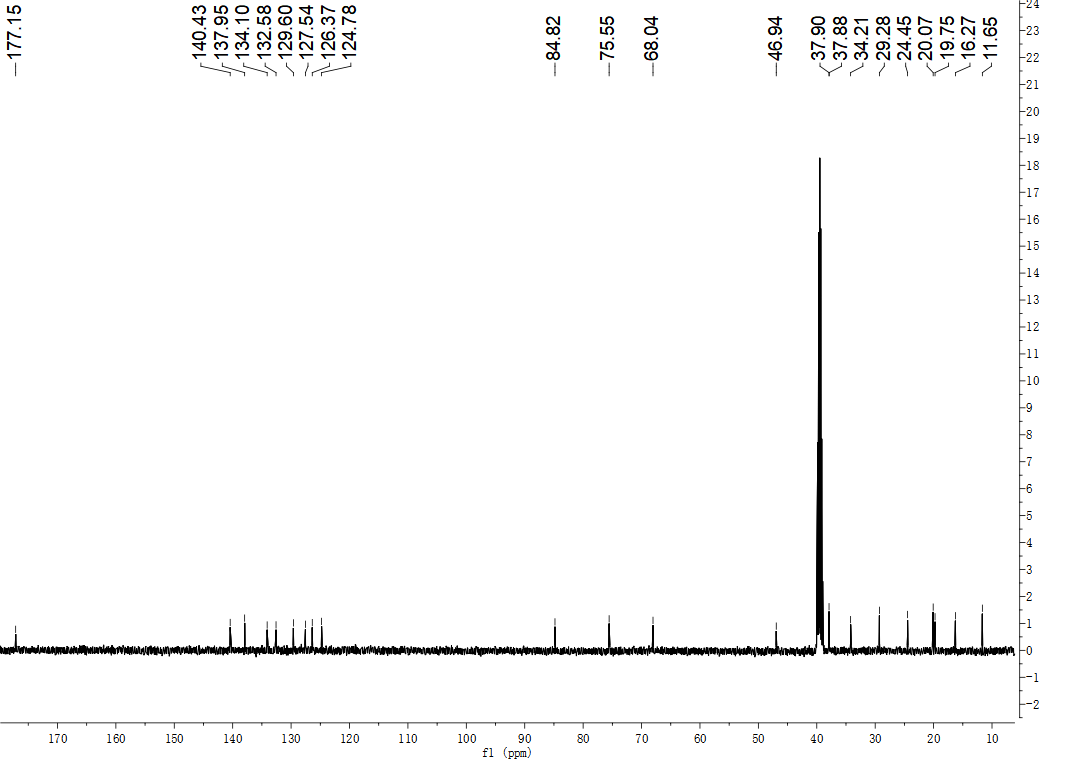


**Figure S2.** ^13^C NMR (125 MHz, DMSO-*d*_6_) spectrum of compound **1**


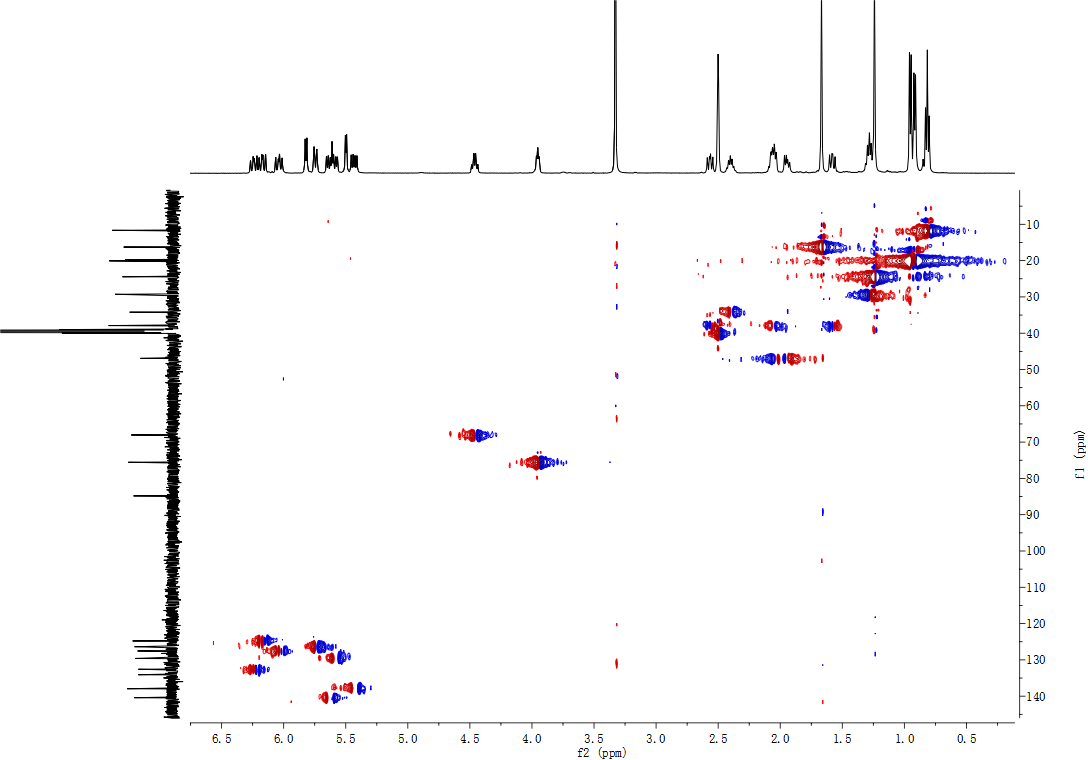


**Figure S3.** HMQC (DMSO-*d*_6_) spectrum of compound **1**


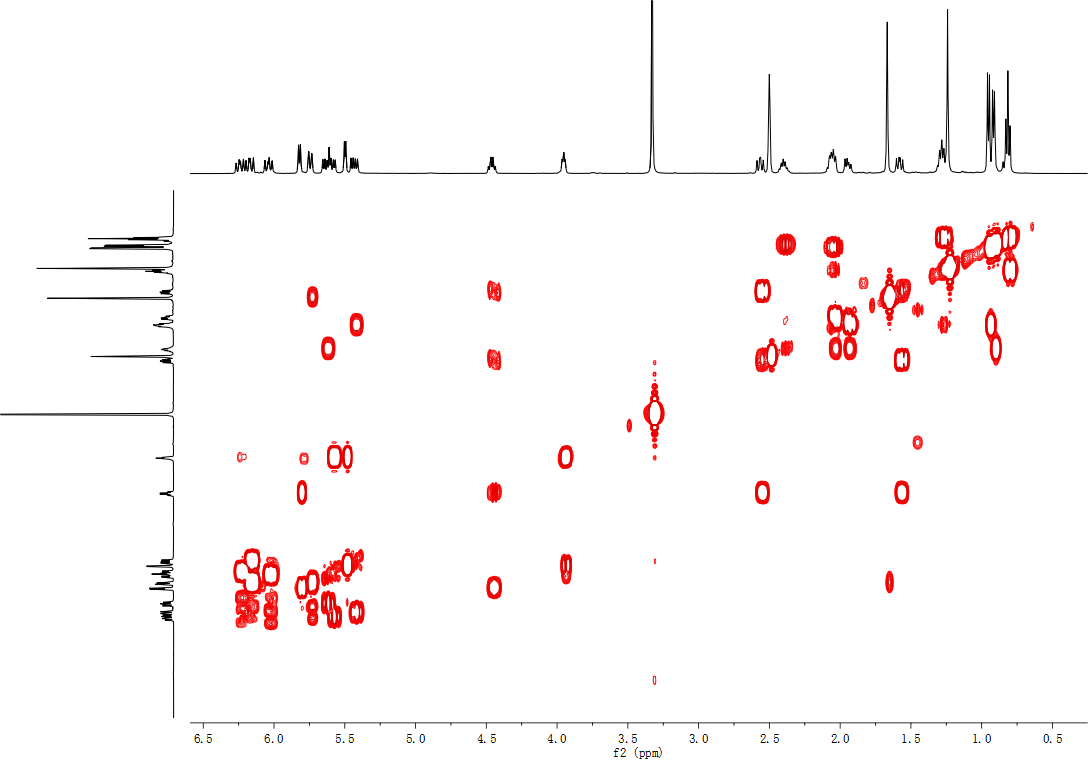


**Figure S4.** COSY (DMSO-*d*_6_) spectrum of compound **1**


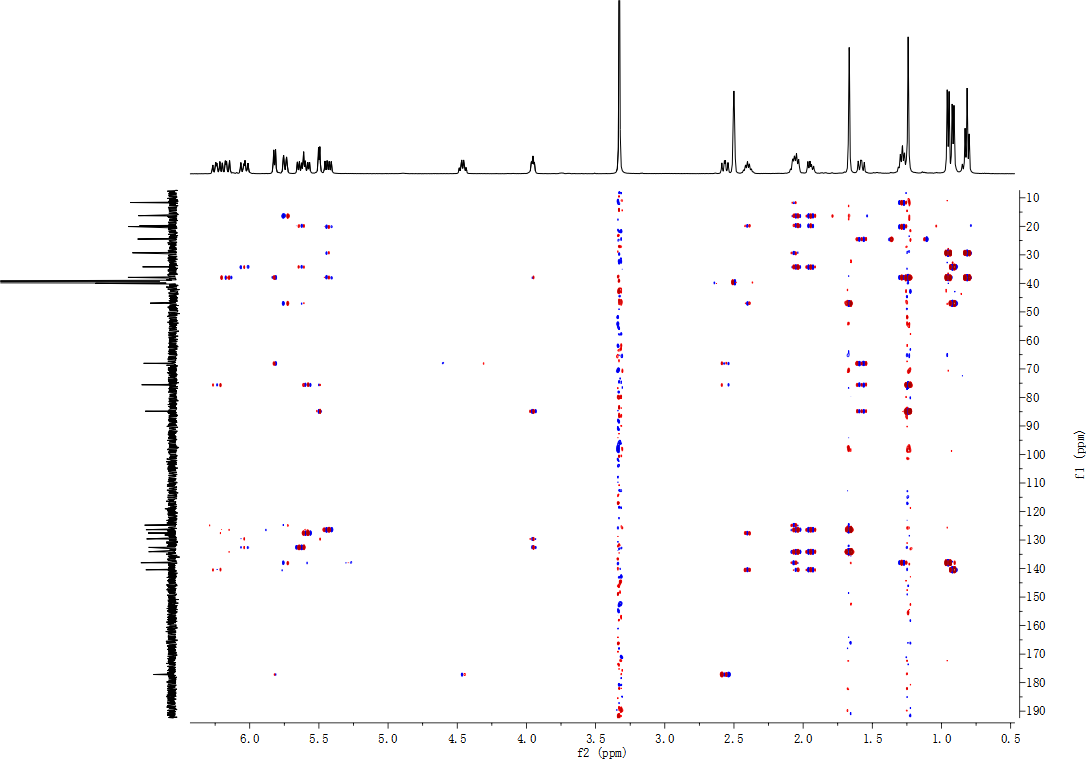


**Figure S5.** HMBC (DMSO-*d*_6_) spectrum of compound **1**


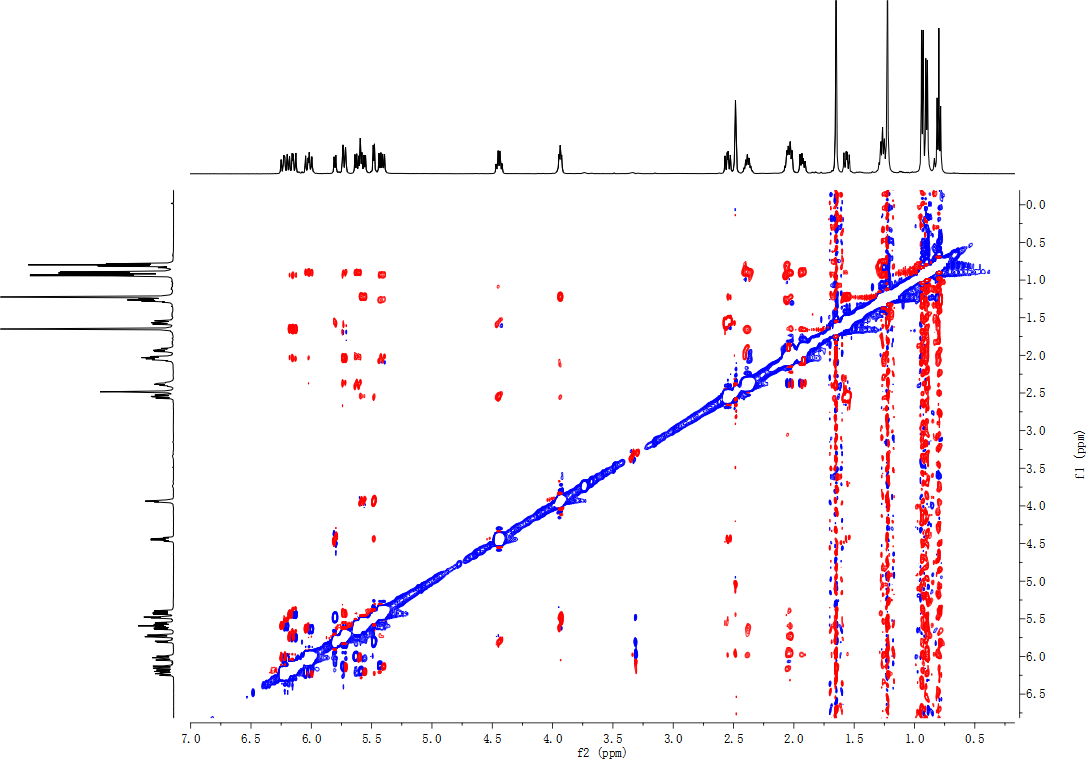


**Figure S6.** NOESY (DMSO-*d*_6_) spectrum of compound **1**

**Figure S7.** HRESIMS spectrum of compound **1**


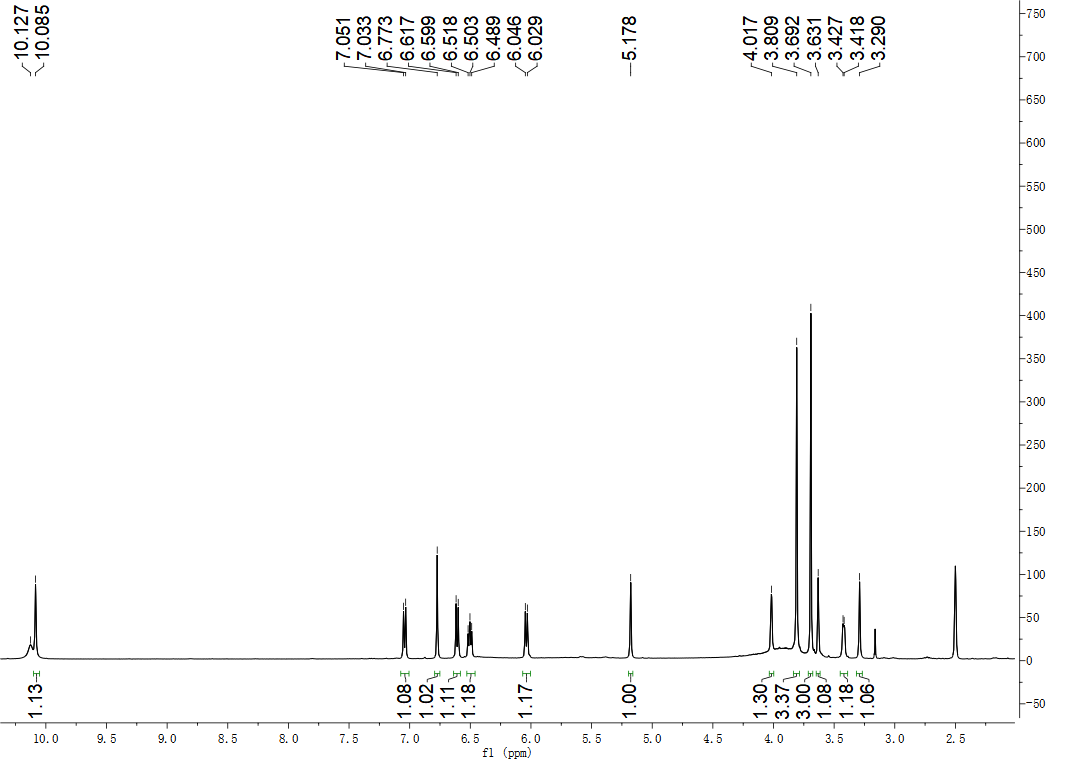


**Figure S8.** ^1^H NMR (500 MHz, DMSO-*d*_6_) spectrum of compound **3**


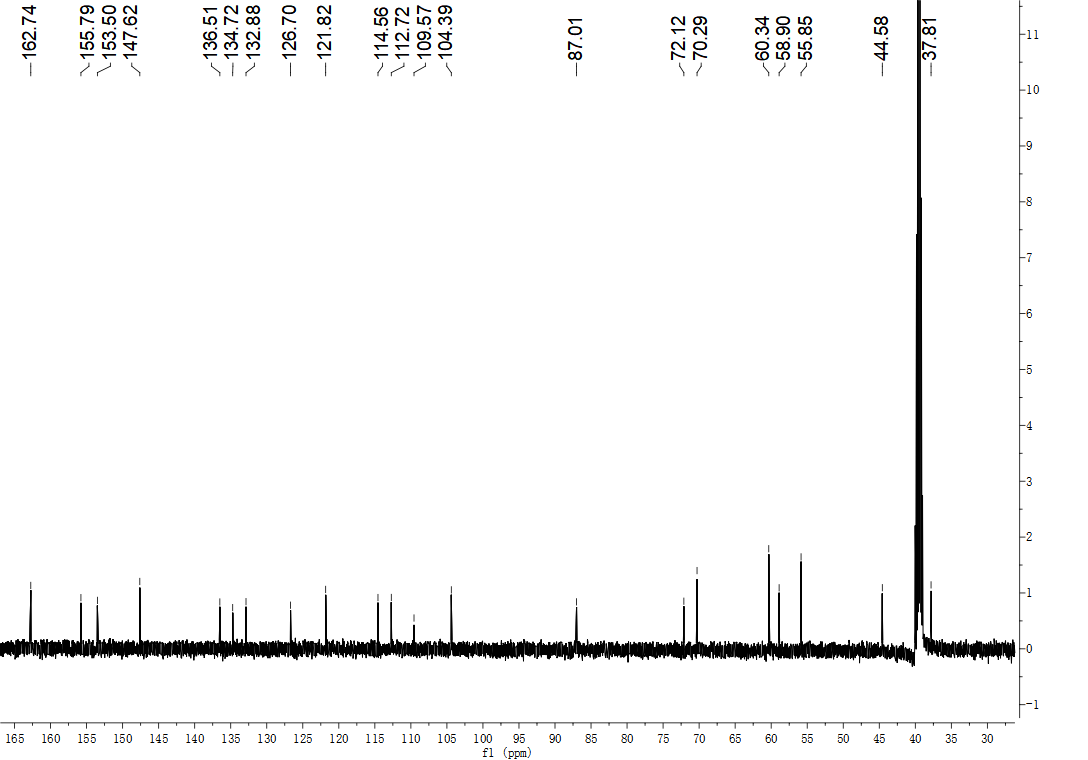


**Figure S9.** ^13^C NMR (125 MHz, DMSO-*d*_6_) spectrum of compound **3**


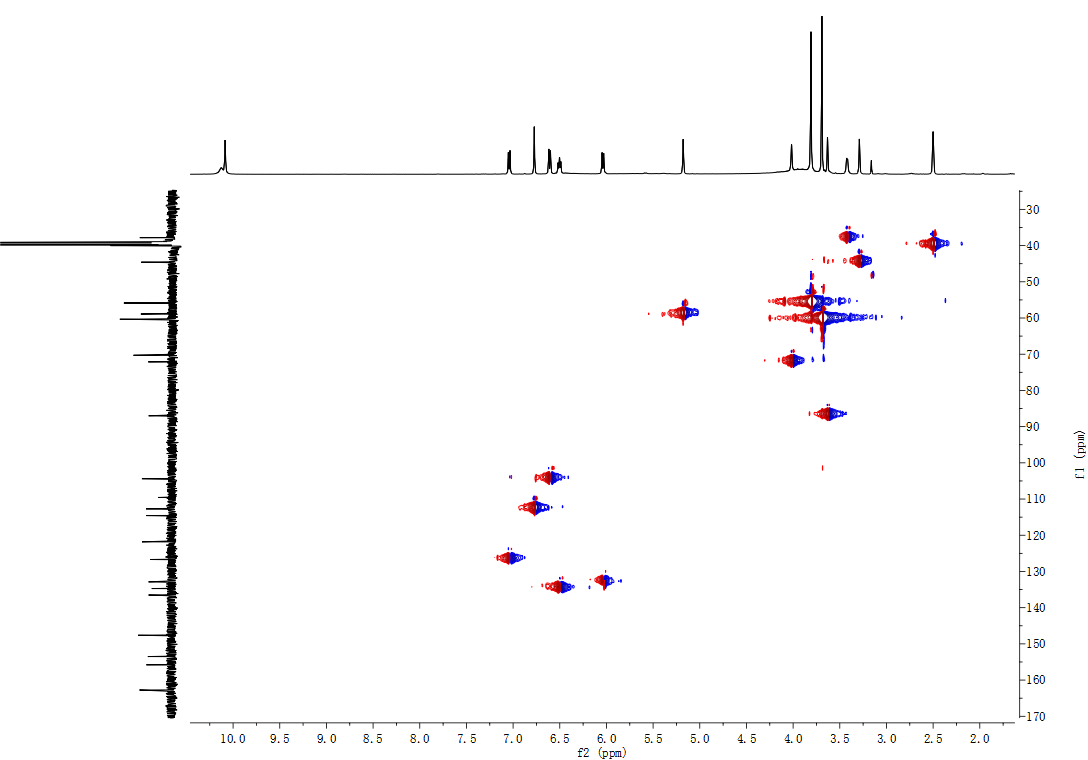


**Figure S10.** HMQC (DMSO-*d*_6_) spectrum of compound **3**


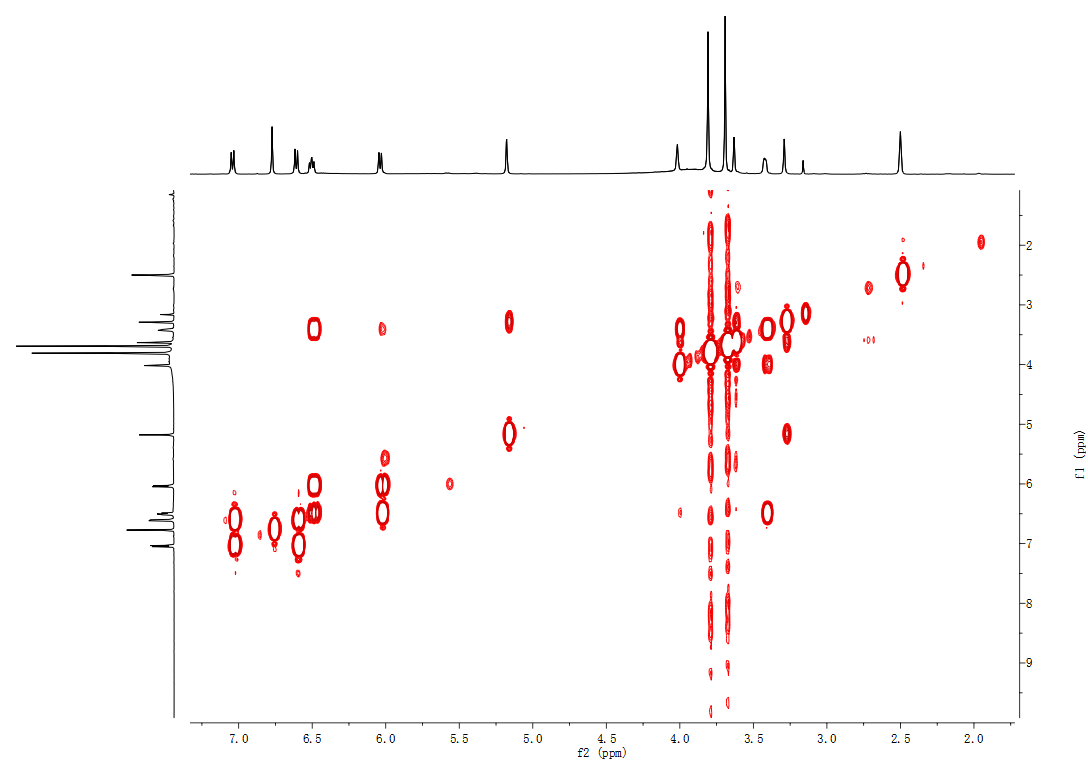


**Figure S11.** COSY (DMSO-*d*_6_) spectrum of compound **3**


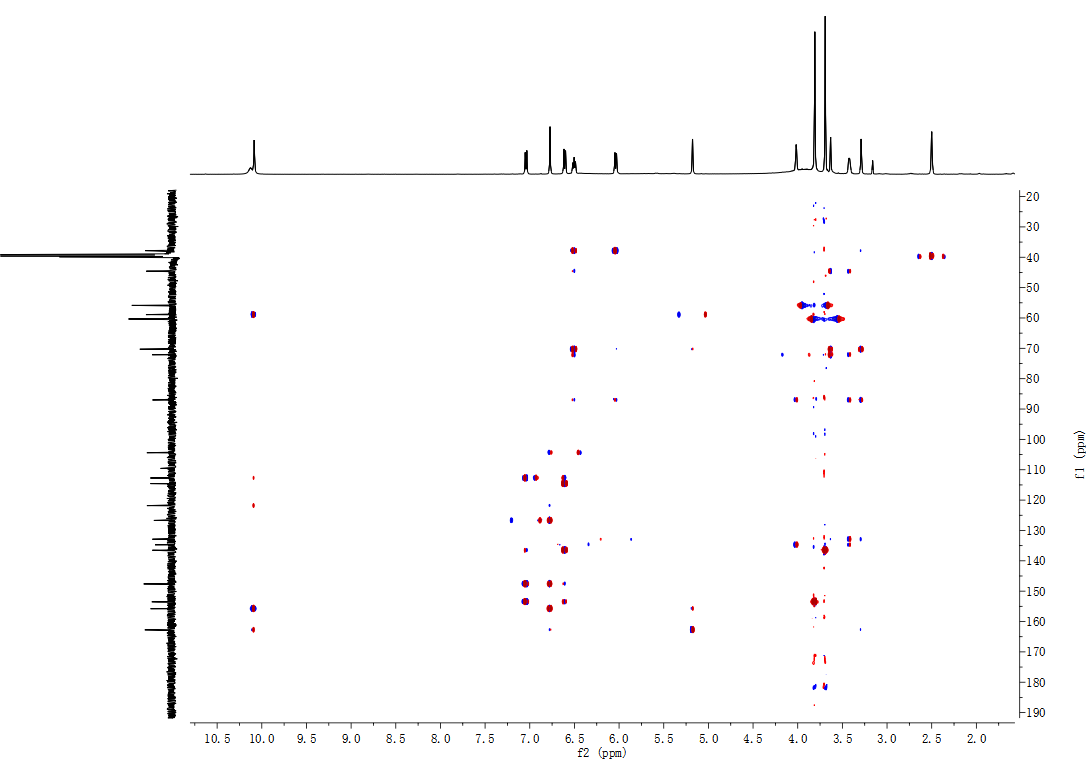


**Figure S12.** HMBC (DMSO-*d*_6_) spectrum of compound **3**


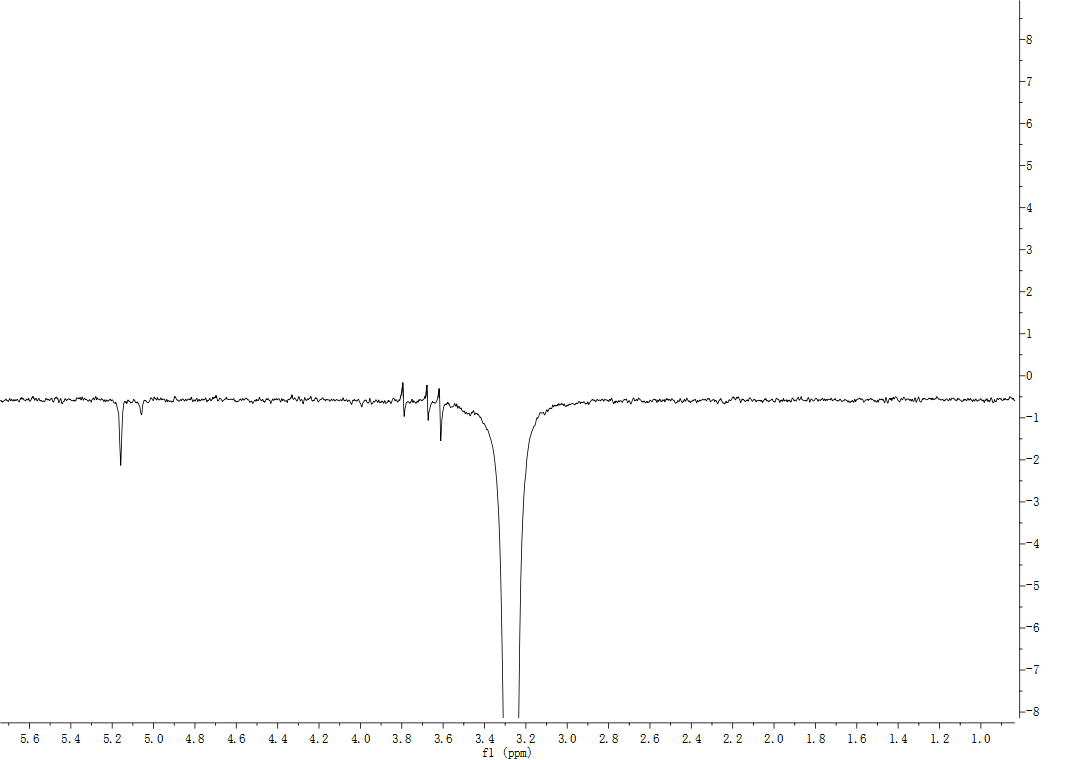


**Figure S13.** 1D NOE (DMSO-*d*_6_) spectrum of compound **3**


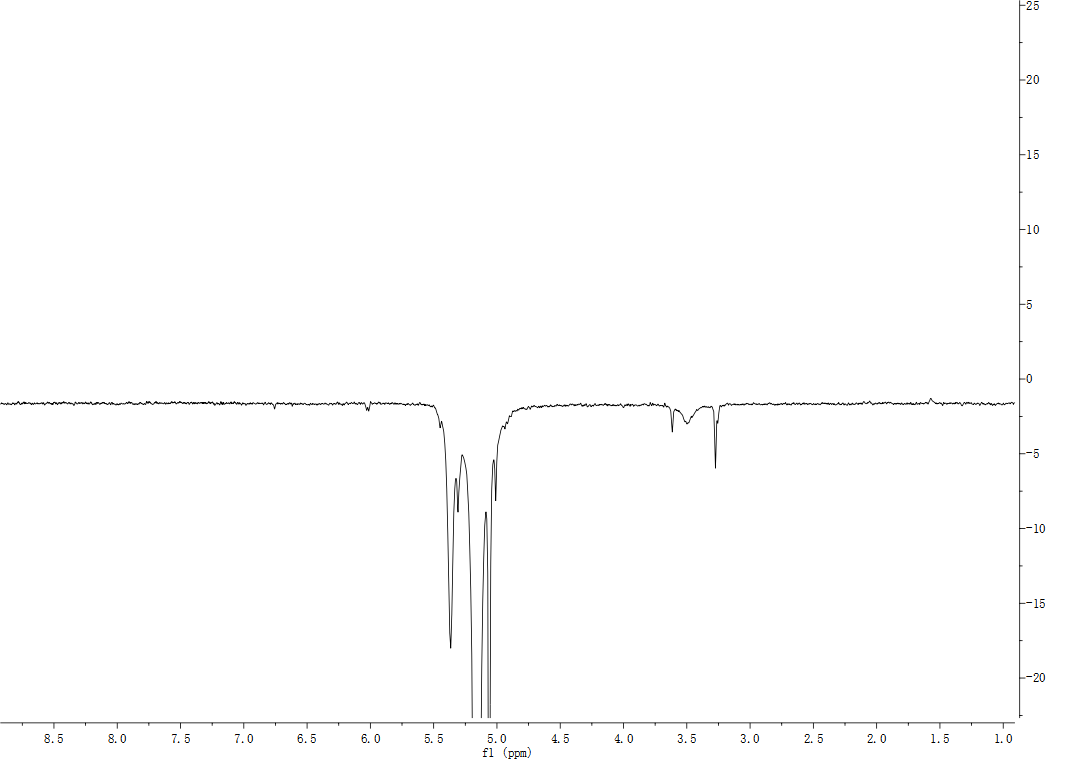


**Figure S14.** 1D NOE (DMSO-*d*_6_) spectrum of compound **3**


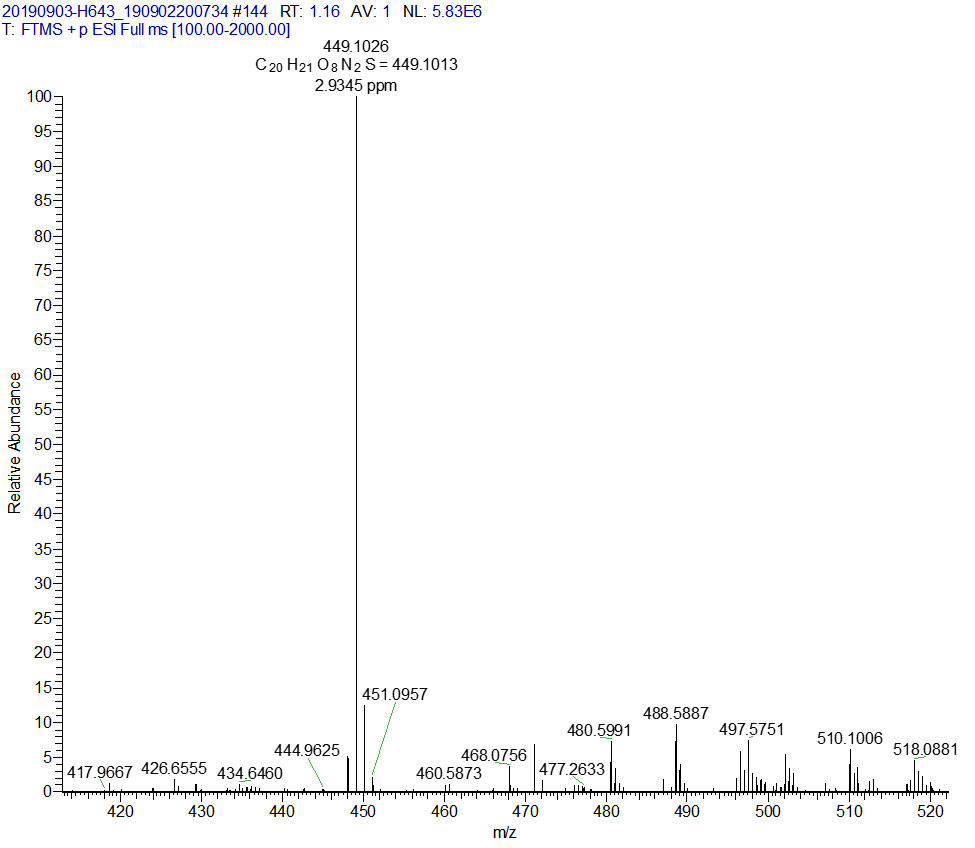


**Figure S15.** HRESIMS spectrum of compound **3**
